# Stigma associated with mental health problems among young people in India: a systematic review of magnitude, manifestations and recommendations

**DOI:** 10.1186/s12888-020-02937-x

**Published:** 2020-11-16

**Authors:** Shivani Mathur Gaiha, Tatiana Taylor Salisbury, Mirja Koschorke, Usha Raman, Mark Petticrew

**Affiliations:** 1 Indian Institute of Public Health- Hyderabad, Public Health Foundation of India, Hyderabad, India; 2 Department of Public Health, Environments and Society, Faculty of Public Health and Policy, London School of Hygiene and Tropical Medicine, London, UK; 3 Centre for Global Mental Health, Institute of Psychiatry, Psychology and Neuroscience, King’s College, London, UK; 4 Health Service and Population Research Department, Institute of Psychiatry, Psychology and Neuroscience, King’s College, London, UK; 5 Department of Communication, Sarojini Naidu School of Arts & Communication, University of Hyderabad, Hyderabad, India

**Keywords:** Stigma, Mental health, Systematic review, India, Youth

## Abstract

**Background:**

Globally, 20% of young people experience mental disorders. In India, only 7.3% of its 365 million youth report such problems. Although public stigma associated with mental health problems particularly affects help-seeking among young people, the extent of stigma among young people in India is unknown. Describing and characterizing public stigma among young people will inform targeted interventions to address such stigma in India, and globally. Thus, we examined the magnitude and manifestations of public stigma, and synthesised evidence of recommendations to reduce mental-health-related stigma among young people in India.

**Method:**

A systematic review and meta-analysis of observational studies was conducted. Nine electronic databases were searched and 30 studies (*n* = 6767) met inclusion criteria.

**Results:**

Most studies (66%) focused on youth training to become health professionals. One-third of young people display poor knowledge of mental health problems and negative attitudes towards people with mental health problems and one in five had actual/intended stigmatizing behavior (I^2^>=95%). Young people are unable to recognize causes and symptoms of mental health problems and believe that recovery is unlikely. People with mental health problems are perceived as dangerous and irresponsible, likely due to misinformation and misunderstanding of mental health problems as being solely comprised of severe mental disorders (e.g. schizophrenia). However, psychiatric labels are not commonly used/understood.

**Conclusion:**

Public education may use symptomatic vignettes (through relatable language and visuals) instead of psychiatric labels to improve young people’s understanding of the range of mental health problems. Recommended strategies to reduce public stigma include awareness campaigns integrated with educational institutions and content relevant to culture and age-appropriate social roles.

**Supplementary Information:**

The online version contains supplementary material available at 10.1186/s12888-020-02937-x.

## Background

Young people, including adolescents and young adults aged 10–24 years [1] are at a critical period in the prevention and treatment of mental health disorders. Globally, an estimated one in five young people experience a mental disorder [2]. Among adults with a mental disorder, 75% report first experiencing a mental disorder during this period [3]. Although public stigma universally prevents people who experience mental health problems (i.e. symptoms that are not sufficient to warrant a diagnosis of a mental disorder) and those with mental disorders from seeking counselling and treatment, [4, 5] the extent and manifestations of such stigma varies across cultures [6, 7]. Public stigma is defined as interrelated ‘problems of knowledge (ignorance), problems of attitudes (prejudice), and problems of behaviour (discrimination)’ [8]. In India too, public stigma is an important factor in the underreported prevalence of mental disorders, [9, 10] with only 7.3% of young people in India reporting a mental disorder and fewer accessing treatment [9].

Mental-health-related public stigma negatively impacts help-seeking by young people to a larger extent than among adults [11–15]. Young people with mental health problems are more likely to experience greater social distance from the public [16]. Additionally, compared to adults, young people do not seek help for mental health problems due to characteristic fears about lack of confidentiality, peer pressure, a desire to be self-reliant, [17] and lack of knowledge to recognize mental health problems [18] or lack of awareness about mental-health-related services [4]. Unsurprisingly, adolescents in a study found it more difficult to disclose their mental health problems compared to young adults [19].

The level of mental-health-related stigma among young people in India remains unknown. Stigma research in the United States, Greece, and Japan [20–22] identifies social distance and discriminatory beliefs related to mental health problems and a systematic review found stigma of mental disorders associated with violence, unpredictability and disability in Latin America and the Caribbean [23]. With the largest young population in the world at 365 million, [24] and a large burden of untreated mental health problems, young people in India will likely face challenges in achieving their social and economic potential. In 2015–16, India’s national mental health survey highlighted that data on such mental-health-related stigma were limited [9]. Reducing public stigma is an aim in India’s national mental health policy, [25] and in April 2017, India passed a law protecting the right to equality and non-discrimination of people with mental illness [26]. Through a systematic review and meta-analysis, this study aims to estimate the magnitude or prevalence of mental-health-related public stigma among a sub-group of the Indian population, i.e. young people aged 10–24 years old belonging to the general population; identify common problems in knowledge, attitude and behaviours associated with mental health; and collate recommendations for reducing mental-health-related public stigma.

## Method

### Eligibility criteria

Studies were included in this systematic review if they assessed public stigma associated with mental health problems among young people (aged 10–24 years) in India. Quantitative and qualitative studies were included if they examined any component of mental-health-related public stigma: knowledge of mental health; attitude towards people with mental health problems; and (intended or actual) behaviour towards people with mental health problems. Studies were excluded if they focused on stigma experienced by people with a diagnosed mental disorder and caregivers, or vulnerable groups at rehabilitation centres, schools for special needs, prisons or shelters, exposed to violence or in conflict zones. These studies were excluded as they involved specific groups for whom explanatory models, quality of life, anticipated or experienced stigma related to personal/lived experiences and previously accessing care or treatment from mental health providers, likely influence knowledge about mental health problems, and attitude and behaviour towards people with mental health problems. Theoretical or methodological studies and protocols for systematic reviews, media articles and social media, policy statements, book reviews, interviews, and lists of books were excluded. No restriction was placed on language of publication or publication date.

### Information sources

Nine databases were searched (PubMed, ADOLEC, CINAHL+, PsycINFO, Scopus, Social policy and practise, Global Health, Web of Science and IndMED). The search was started in October 2014, and the last search in all databases was conducted in September 2018. Results were managed in EndNote X9 [27].

Methods and findings are reported according to the Preferred Reporting Items for Systematic reviews and Meta-analyses (PRISMA) guidelines, see Supplementary material [28]. The search strategy is presented in Table 1.

**Table 1:** Search strategy for studies of youth mental-health-related stigma in India

Category	Search terms
Stigma	(stigma or knowledge or awareness or myth or stereotyp^a^ or attitude or prejudice or negativ^a^ or discriminat^a^ or exclusion or “social distance” or “intended behaviour” or avoid^a^ or victim^a^ or violen^a^ or isolat^a^)
AND	
Mental health	(mental OR psychiatr^a^ OR psychol^a^ OR anxiety OR panic OR bipolar OR “personality disorder” OR depression OR dissociative OR alcohol^a^ OR dependency OR schizophreni^a^ OR mania OR “learning disability” OR obsessive AND compulsive OR self AND harm OR self-harm OR paranoi^a^ OR phobia^a^ OR “post traumatic stress” OR insomnia^a^ OR suicid^a^ OR addicti^a^ OR bereave^a^ OR “attention deficit” OR body AND dysmorphic OR delirium OR delusion^a^ OR hallucinat^a^ OR hyperactiv^a^ OR delinquen^a^ OR aggress^a^ OR “substance use” OR “substance abuse”)
AND	
India	(India)

^a^Symbol of truncation in order to search keywords with varying endings and plural forms

### Study selection

The first author conducted this search across all databases and reviewed all studies based on the eligibility criteria, by reading all titles; next, by reading selected abstracts; and lastly, by reading the full text and references. Wherever there was incomplete information to include a study, it was moved to the next stage. If two or more articles on the same target population were found, the sample sizes and method were compared to confirm that the population studies were the same, and the most relevant article pertaining to eligibility criteria mentioned above, was retained for analysis. In the event that it was unclear if an article met review inclusion criteria, the first author discussed the article with the senior author.

### Data extraction

The framework for data extraction included the following study characteristics: year of publication, sample size, location, % females, participant age, independent variables, dependent/ outcome variables corresponding to knowledge, attitude and actual/ intended behaviour components of mental-health-related public stigma reported. We extracted data from all studies where authors self-identified that they measured knowledge, attitude and actual/ intended behaviour (components of public stigma). In addition, we reviewed abstracts followed by full-texts of studies found using our search strategy, and based on research question, individual measures and results corresponding to each public stigma component, data were extracted. Ultimately, a variety of measures were used to assess each of these stigma components. The risk of bias in included studies was assessed using the National Institutes of Health Quality Assessment Tool for Observational Cohort and Cross-Sectional Studies [29]. Qualitative narratives about knowledge, attitude and behaviour related gaps in public stigma and recommendations to reduce public stigma were collated from both qualitative and quantitative studies.

### Summary measures

The principal measures used in the primary studies include percentages, means (standard deviation), differences between means, and levels of significance (*p*-values).

### Synthesis and reporting

First, demographic information of participants was extracted from all survey studies as per review objectives. Second, heterogeneity across studies assessed through I^2^ values determined if a meta-analysis of public stigma levels was appropriate. Similar to studies on prevalence of mental-health-related public stigma from Greece [21] and the United States, [20] we calculated such prevalence among youth. Public stigma levels were plotted by pooling study-wise percentage data on agreement with key statements related to knowledge, attitude and behaviour. If Standard Error (SE) was not reported by a study, the following formula was used: SE = $\sqrt{\mathrm{p}\left(1-\mathrm{p}\right)}/\mathrm{n}$ and 95%CI = p ± 1.96 X SE; where, *p* = percentage of participants agreeing with items/ statements displaying poor knowledge, negative attitudes and stigmatising actual or intended behaviours and CI = Confidence Interval. If a study reported multiple items corresponding to each public stigma component, then the item with the lowest (stigmatizing) percentage was included. For example, within the attitude domain of public stigma, if a study reported different percentages of participants who believed that persons with mental illness ‘lack will power,’ ‘are to blame’ and ‘can’t handle responsibilities,’ then the lowest percentage was plotted. Review Manager software (Version 5.3.5) was used to conduct the meta-analysis [30]. Random-effects models were generated to calculate the pooled percentage of public stigma as studies were likely from different regions of India, with variations in population, subject selection methods and measures.

Third, a narrative synthesis [adapted from existing Economic and Social Research Council (ESRC) guidance] [31] as per study objectives was used to collate and group qualitative findings corresponding to common conceptual gaps and perceptions related to each public stigma component (knowledge, attitude or behaviour) and recommendations to reduce stigma. Gaps were presented in descending order of frequency (number of times a theme was repeated across multiple studies) and importance (theme was included in the study discussion).

## Results

**Fig. 1:**
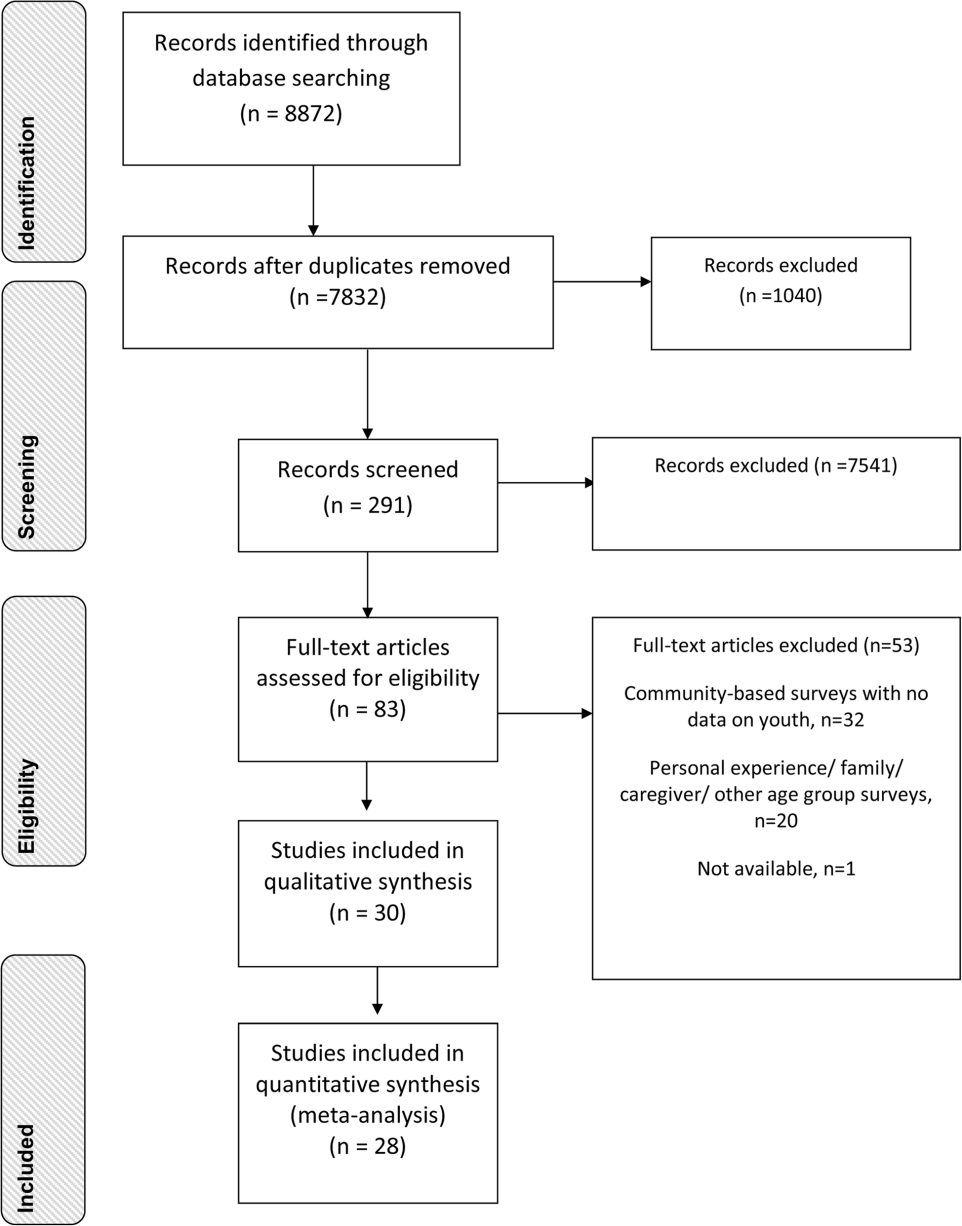
PRISMA Flow diagramme for youth stigma associated with mental health in India

Thirty studies were selected from 8872 articles in this review (Fig. 1). After removing 1040 duplicate articles, 7832 titles were screened based on the inclusion criteria. Next 291 abstracts were reviewed, of which 83 full-text studies were identified. One full-text article was unavailable [32].

### Study characteristics

Twenty-eight quantitative studies [33–60] and two qualitative research studies [61, 62] were included in this review. A summary of data from these studies on sample size, age, gender, location (rural or urban), and outcomes related to public stigma domains: knowledge (K), attitude (A) and actual/ intended behaviour (B) is presented in Table 2.

**Table 2:** Summary of study characteristics from youth-assessments of stigma in India

**(Author, Year)**	**Aspect of mental health**	**Sample size, Site: Urban(U)/ Rural(R)**	**Age (mean or range)**	**% of female**	**Location**	**Participant profile**	**Stigma component/s [Knowledge (K); Attitude (A); Behaviour (B)]**	**Knowledge (K)**	**Attitude (A)**	**Actual or intended behaviour (B)**
Abraham et al., 2014 [33]	Nomophobia - fear of being without or being unable to use one’s phone	200 (U)	18–23	NR	Karnataka	College students	K	89% had poor knowledge	–	–
Aggarwal et al., 2016 [34]	Mental illness	289 (U)	20.5	54.7	Delhi	Medical, psychology & other college students	KAB	Causes of mental illness - genetic, brain damage; God’s punishment, stress, biological factors and physical and sexual abuse	Mentally ill tend to be ‘mentally retarded’ and of low intelligence; need prescription drugs to control; marriage or psychotherapy can successfully treat mental illness	Majority would not maintain a friendship and would feel ashamed if they were related to a person with mental illness
Ahuja et al., 2017 [35]	Mental illness	50 (U)	18–21	54	Delhi-National Capital Region	College students of History, English, Business and Journalism	A	–	15% negatively labelled mental health problems and 77% used negative descriptions such as ‘stubborn, untidy and unclean’	–
Bell et al., 2010 [36]	Mental illness	106 (U)	20/19–23	57	Maharashtra	Pharmacy	AB	–	Perception that people will never recover	18.75+/− 3.57
Bell et al., 2008 [37]	Epilepsy	106 (U)	21/19–24	58	Maharashtra	Pharmacy	A	–	People with depression will never recover and people with schizophrenia will never recover	–
Bhise et al., 2016 [38]	Mental illness	94 (U)	21.9 (0.7); 20.6 (0.8)	44.6;88.3	Maharashtra	Psychiatry and physiotherapy	A	–	3.3–3.4 (0.94, Kruskal Wallis *p* = 0.3)	–
Chawla et al., 2012 [39]	Mental illness	210 (U)	20.2 (1.63)	30.9	NR	Undergraduate medical students	KA	Mental illness - occurs among people who have excessive emotions (66%) or are lonely (52%); is caused by past sins/evil spirits (16%); is not treatable (7%).	46% felt fear, anger or hatred when they met a psychiatric patient	
D’Sa et al., 2016 [40]	Substance use	487 (U)	< 18	NR	Mangalore, Karnataka	School students and college students	K	58% knew where people can access alcohol	–	–
Etzersdorfer et al., 1998 [41]	Suicide	100 (U)	18.4	51.5	Chennai, India	Medical students	A	–	70% said suicide by someone most near was cowardly; 37% said it was deliberate	18% would likely commit suicide because they had a mental illness
Gulati et al., 2014 [42]	Mental illness	135 (NR)	20.3	57.3	North India	Medical students (yr. 1 and 2) and interns - upper middle class and middle class	KA	29–65% felt that people with mental illness are easy to recognize, and are different from patients suffering from other illness	68% felt that persons with mental illness should not be treated in the same hospital with people with physical illness. 65–75% prefer locking such patients.	–
Hiremath & Wale, 2017 [43]	Adjustment problems	100 (U)	22–25	70	Tumkur, Karnataka	Third-year B.Sc. Nursing students	KA	15% had poor knowledge about adjustment problems	20% had negative attitudes regarding adjustment problems	
Joshi et al., 2012 [44]	Epilepsy	798 (U)	14–16	33.2	Bareilly, Uttar Pradesh	School students	KAB	Cause of epilepsy: 36% did not know that epilepsy is a brain disorder. 5% believed that epilepsy is infectious. 69% felt that epilepsy can be cured. 4% felt it can be treated by a spiritual healer. Perceived causes for epilepsy: inherited (71%), non-vegetarian diet (49%), evil spirits (5%).	40% believed that average IQ of an epileptic patient is less than a normal person. Most (89%) of the students felt pity/sad for an epileptic patient, and 37% of them thought that an epileptic patient is dangerous. 72% thought that children with epilepsy should study in a special school.	During a seizure, 51.5% of the students would take the person to the hospital, 23.43% would throw water on the person and 22.69% would make the person smell a shoe or an onion.
Kalra, 2012 [61]	Mental illness	11 (U)	NR	NR	Mumbai, Maharashtra	Psychiatry trainees	A	–	Society stereotyping psychiatrists as ‘mad doctors’ Other medical colleagues do not take them seriously and they felt ‘stigmatized along with the psychiatric patients’	–
Kodakandla et al., 2016 [45]	Mental illness	176 (U)	23.2 years (1.06)	81.25	Hyderabad, Telangana	Participants wereInterns, who completed their psychiatry rotation	KA	31% believed that psychological illness is unlikely to becured regardless of the treatment. 76% believed that psychological disorder is recurrent. 68% were of the opinion that people who have once received psychologicaltreatment are likely to need further treatment in the future.	76% believed that a mentally ill person is more likely to harm others. 62% believed that it may be difficult for mentally ill patients to follow social rules and that they are less likely to function well as parents (63%). 82% believe that mentally ill patients should have a job with minor responsibilities. 79% felt that behaviour of patients with psychologicaldisorder is unpredictable.	
Madhan et al., 2012 [46]	Mental illness	212 (U)	NR	NR	Guntur, India	Dental students	A	–	Regard was the highest for persons with intellectualdisability, followed by acute mental illness, and substance misuse.	–
Mahto et al., 2009 [47]	Mental illness	100 (U)	18–35	50	Ranchi, Jharkhand	postgraduate department college	A	–	Females had more neutral attitudes compared to males, although no significant difference overall.	–
Mehrotra et al., 2013 [62]	Mental illness	536 (NR)	21(2.3)/ 17–30	59	NR	Graduate and undergraduate college students	K	Mental health defined positively, although cognitive functioning was stressed.	–	–
Nebhinani et al., 2017 [48]	Suicide	205 (U)	21.9	46	Rohtak, Haryana	Final year medical students	A	–	23% perceived that people with serious suicidal intent do not talk about it. Suicide as attention-seeking.	–
Nebhinani et al., 2013 [49]	Substance use	192 (U)	16.57 (1.63); 19.49 (1.24)	49 and 38%	Chandigarh	College and school students	KA	More college studentsconsidered substance related harm as temporary (7%vs. 1%); 26% considered no treatmentfor substance use.	15% had negative attitude towards substance abusers (labelled them ‘bad people’and added that they should not be helped). and 81% felt that subjects may quit substance with willpower, despite a longer duration of intake.	–
Poreddi et al., 2016 [50]	Mental illness	271 (U)	20.9 (1.7)	80.9	Bangalore, Karnataka	Medical and nursing undergraduates after a psychiatry course	A	–	People with mental illness - should have limited input in to deciding medication to be used (44%); can’t handle too much responsibility (41%).	–
Poreddi et al., 2017 [51]	Mental illness	322 (U)	19.57–20.87	83.9	Bangalore, Karnataka	Medical and nursing undergraduates after completing psychiatry course	AB	–	Medical students reported better attitudes than nursing students regarding stereotyping, restrictiveness, benevolence and pessimistic prediction. Nursing students had better attitude regarding separatism.	Stigmatization among medical students 8.37 ± 2.81) and 9.27 ± 2.48) among nursing students
Poreddi et al., 2015 [52]	Mental illness	116 (U)	20.96 (0.90)	98.3	NR	Nursing undergraduate	A	–	80% said people with mental illness are unpredictable. 71% said they cannot handle too much responsibility, 84% felt they are more likely to commit offences or crimes and 44% believe they are more likely to be violent.	–
Prasad & Theodore, 2016 [53]	Mental illness	400 (U)	NR	82.75	Bangalore, Karnataka	B.Sc. nursing students	K	70% had inadequate knowledge of human rights related to mental illness	–	–
Ram et al., 2017 [54]	Suicide	339 (U)	17–31 (21.80 ± 2.18)	68.7	Mysuru, Karnataka	Undergraduate, postgraduate and interning medical students and paramedical students	KA	36% were unable to identify symptoms of depression; 64% felt that talking about suicide increases risk of suicide, 62% more men commit suicide than women; 65% happens to few people; 52% of people with depression need to be hospitalized.	45.42% would not disclose suicidal ideation; 49.55% - people with mental illness change their mind quickly	
Roy et al., 2017 [55]	Substance use	379 (U)	13.6	NR	Patiala, Punjab	Nr	KA	19% did not know that alcohol is a drug. 22% assumedthat smaller doses of alcohol do no harm.	8% expected alcohol to improvetheir sexual activity.	–
Shanthi et al., 2015 [56]	Substance use	100 (U)	14–17	0	Mangalore, Karnataka	School students	K	Regarding alcoholism and its effects: 80% had average knowledge, 17% hadpoor knowledge and 3% had good knowledge	–	–
Sureka et al., 2016 [57]	Epilepsy	411 (U)	NR	NR	Jaipur, Rajasthan	Nursing and medical students	KAB	Causes of epilepsy: epilepsy is a mentalillness (27–40%); birthdefect and blood disorder (25%); family history (21–39%) and supernatural power (5%) Symptoms of epilepsy: loss ofconsciousness and convulsions (55–58%) Treatment by allopathic medicine, followed by ayurvedic and homeopathic (40–50%)	Epilepsy is a hindrance in life (50–76%). An “epileptic person” should not marry (25–33%). Among both groups, most participants would like to play/study with epileptic child. 23% thoughtepileptics have committed sins in past life.	During an epileptic attack, majority would take a patient to the hospital and 16%in one group would put water/ shoe/ onion on the person’sface.
Thakur & Olive, 2016 [58]	Nomophobia - fear of being without or being unable to use one’s phone	100 (U)	NR	NR	Jalandhar, Punjab	College students of nursing, technology and engineering	K	68% had poor knowledge of nomophobia	–	–
Thomas et al., 2015 [59]	Substance use	60 (U)	13–15	50	Malkapur, Maharashtra	School students	K	23% had poor knowledge regarding substance use	–	–
Vijayalakshmi et al., 2013 [60]	Mental illness	268 (U)	NR	100	Bangalore, Karnataka	Nursing and management students	KAB	Stereotyping sub-scale: 22% (*n* = 33) of the nursing and 12% (*n* = 14) BBM students felt that people with mental illness cannot be easily identified by their behaviour. People with mental illness have a lower IQ according to 35.8% nursing students (*n* = 95) and 78.3% BBM students (*n* = 26). More nursing (*n* = 92, 62.2%) than BBM students (*n* = 34, 28.3%) accepted that ‘everyone faces the possibility of becoming mentally ill’.	Separatism sub-scale: more nursing students (*n* = 128, 86.5%) than BBM students (*n* = 86, 71.7%) would not move out of the community if a mental health facility was set up. More nursing (n = 26, 17.6%) than BBM students (*n* = 15, 12.5%) disagreed that people with mental illness are violent and dangerous. 76.3% (*n* = 113) of nursing students compared to 52.5% (*n* = 63) of BBM students agreed that the ‘mentally ill should be able to have children’. More nursing (*n* = 69, 46.7%) than BBM students (*n* = 21, 17.5%) agreed that ‘people with mental illness can hold a job	More nursing students (*n* = 50, 33.8%) than BBM students (*n* = 38, 31.6%) felt that the ‘mentally illshould not disclose their illness’. Both nursing (*n* = 112, 75.7%) and BBM students (*n* = 82, 68.3%) agreed that they ‘should not laugh at the mentally ill’

Data from 6767 young people were included. Few studies included young people below 18 years of age [40, 44, 49, 54, 56, 59, 62, 63] and studies varied by the proportion of females (33–100%). Twenty studies assessed stigma among college students who were health professionals-in training, i.e. those studying medical, psychiatry, dental, pharmacy and nursing [34, 36–39, 41–43, 45, 46, 48, 50–54, 57, 60, 61]. Three studies included college students pursuing other disciplines [35, 58, 60]. Four secondary school-based studies were found [44, 55, 56, 59].

### Outcomes measured

One-third of all studies assessing mental-health-related stigma among youth reported on attitude [35, 37, 38, 45–48, 50, 52, 61]. Eight studies assessed knowledge related to mental illness, [33, 39, 40, 53, 56, 58, 59, 62] seven studies assessed knowledge and attitude towards people with mental illness [41–43, 49, 54, 55, 60] and two studies focused on attitude and intended behaviour towards people with mental illness [36, 51]. Only three studies assessed stigma comprehensively, across all components: knowledge, attitude and intended/actual behaviour [34, 44, 57]. As presented in the summary of study characteristics in Table 2, 16 out of 30 studies focused on stigma associated with mental illness (broadly defined, with no specific disorders included or excluded). The remaining studies were divided among epilepsy, phobia, suicide, and substance use (as specific disorders). Some of these studies included surveys with specific items/ scales measuring anxiety, panic disorder, bipolar disorder, depression, schizophrenia, and stress which are specific disorders/conditions. Stigma-related outcomes were measured using the Guttman social distance scale, [34, 51] Attitude To Psychiatry-29 [42, 45] and 30, [38] SUIATT questionnaire, [41] Opinions about Mental Illness, [42, 60] Beliefs towards Mental Illness scale, [45] and the Attitude Scale for Mental Illness (ASMI) [34, 51]. Other studies reported developing their own survey questionnaires. No study used a vignette-based survey to assess recognition of signs and symptoms of mental illness.

### Risk of bias within studies

Among quantitative studies, six studies were of good quality, and 11 each were of fair quality and poor quality (Table 3). One-third of all quantitative studies (*n* = 28), reported how the study population was selected: seven studies used purposive sampling, [33, 36–40, 48] and one study each used stratified random sampling, [34] two-stage random sampling, [53] and simple random sampling [59]. The rationale and calculation for sample size were presented in only one study [40]. The rate of participation was more than 50% in nine studies [37, 38, 40–42, 49–52] and other studies did not report participation rates. Only 15 studies (53%) used varied validated instruments to measure stigma [34, 36–38, 41, 42, 45, 46, 50–52, 54, 60]. Five studies adjusted mental health stigma outcomes with potential confounding variables [33, 34, 51, 52, 58].

**Table 3:** Youth Stigma in India: Risk of bias assessment (*Y* Yes, *N* No, *NA* Not applicable, *NR* Not reported)

**Studies (1–10) ➔**	**Abraham et al., 2014** [33]	**Aggarwal et al., 2016** [34]	**Ahuja et al., 2017** [35]	**Bell et al., 2010** [36]	**Bell et al., 2008** [37]	**Bhise et al., 2016** [38]	**Chawla et al., 2012** [39]	**D’Sa et al., 2016** [40]	**Etzersdorfer et al., 1998** [41]	**Gulati et al., 2014** [42]
1. Was the research question or objective clearly stated?	Y	Y	Y	Y	Y	Y	Y	N	Y	Y
2. Was the study population clearly specified and defined?	Y	Y	N	Y	Y	Y	N	Y	Y	Y
3. Was the participation rate of eligible persons at least 50%?	NR	Y	NR	Y	Y	Y	NR	Y	Y	Y
4. Were all subjects selected or recruited from the same or similar populations?	N	Y	N	Y	Y	Y	Y	Y	N	Y
5. Was a sample size justification, power description, or variance and effect estimates provided?	N	N	N	N	N	N	N	Y	N	N
6. Were exposure(s) of interest measured prior to the outcome(s) being measured?	N	N	Y	N	Y	Y	Y	Y	Y	Y
7. Was the timeframe sufficient so that one could reasonably expect to see an association between exposure and outcome if it existed?	NR	NR	NR	NR	NA	NA	NR	NA	NR	NR
8. For exposures that can vary in amount or level, did the study examine different levels of the exposure as related to the outcome?	Y	Y	N	Y	Y	N	Y	NR	Y	Y
9. Were exposure measures clearly defined, valid, reliable, and implemented consistently across all participants?	Y	N	Y	N	N	N	N	NR	N	Y
10. Was the exposure(s) assessed more than once over time?	N	N	N	N	N	N	N	N	N	N
11. Were the outcome measures clearly defined, valid, reliable, and implemented consistently across all participants?		N	Y	Y	N	Y	N	N	Y	Y
12. Were outcome assessors blinded to the exposure status of participants?	Y	N	N	N	N	N	N	N	N	N
13. Was loss to follow-up after baseline 20% or less?	NA	NA	NA	NA	Y	Y	NA	Y	NA	NA
14. Were key potential confounding variables measured and adjusted statistically for their impact on the relationship between exposure(s) and outcome(s)?	Y	Y	N	N	N	N	N	N	N	N
**Quality rating [Good (G); Fair (F); Poor (P)]**	**F**	**G**	**P**	**F**	**G**	**G**	**P**	**F**	**F**	**G**
**Studies (11–21)➔**	**Hiremath & Wale, 2017** [43]	**Joshi et al., 2012** [44]	**Kalra, 2012** [61]	**Kodakandla et al., 2016** [45]	**Madhan et al., 2012** [46]	**Mahto et al., 2009** [47]	**Mehrotra et al., 2013** [62]	**Nebhinani et al., 201** [49]	**Nebhinani et al., 2017** [48]	**Poreddi et al., 2016** [50]
1. Was the research question or objective clearly stated?	Y	Y	Y	Y	Y	N	Y	Y	Y	Y
2. Was the study population clearly specified and defined?	Y	Y	Y	Y	Y	N	Y	Y	Y	Y
3. Was the participation rate of eligible persons at least 50%?	N	NR	Y	NR	NR	NR	Y	Y	NR	Y
4. Were all subjects selected or recruited from the same or similar populations?	Y	Y	Y	Y	Y	N	Y	Y	Y	Y
5. Was a sample size justification, power description, or variance and effect estimates provided?	N	N	N	N	N	N	N	N	N	N
6. Were exposure(s) of interest measured prior to the outcome(s) being measured?	Y	Y	N	Y	N	Y	N	N	N	N
7. Was the timeframe sufficient so that one could reasonably expect to see an association between exposure and outcome if it existed?	NR	NA	NA	NA	NR	NR	NR	N	NR	N
8. For exposures that can vary in amount or level, did the study examine different levels of the exposure as related to the outcome?	Y	NR	NR	Y	N	Y	N	N	Y	N
9. Were exposure measures clearly defined, valid, reliable, and implemented consistently across all participants?	N	N	N	Y	N	N	Y	N	N	N
10. Was the exposure(s) assessed more than once over time?	N	N	NA	N	N	N	N	N	N	N
11. Were the outcome measures clearly defined, valid, reliable, and implemented consistently across all participants?	N	N	N	Y	Y	Y	Y	Y	Y	Y
12. Were outcome assessors blinded to the exposure status of participants?	N	N	N	N	N	N	N	N	N	N
13. Was loss to follow-up after baseline 20% or less?	NA	NA	NA	NA	NA	NA	NA	NA	NA	NA
14. Were key potential confounding variables measured and adjusted statistically for their impact on the relationship between exposure(s) and outcome(s)?	N	N	N	N	N	N	N	N	N	N
**Quality rating [Good (G); Fair (F); Poor (P)]**	**F**	**P**	**P**	**G**	**P**	**P**	**F**	**F**	**F**	**F**
**Studies (21–30)➔**	**Poreddi et al., 2017** [51]	**Poreddi et al., 2015** [52]	**Prasad & Theodore, 2016** [53]	**Ram et al., 2017** [54]	**Roy et al., 2017** [55]	**Shanthi et al., 2015** [56]	**Sureka et al., 2016** [57]	**Thakur & Olive, 2016** [58]	**Thomas et al., 2015** [59]	**Vijayalakshmi et al., 2013** [60]
**1. Was the research question or objective clearly stated?**	**Y**	**Y**	**Y**	**Y**	**Y**	**Y**	**Y**	**Y**	**Y**	**Y**
**2. Was the study population clearly specified and defined?**	**Y**	**Y**	**Y**	**Y**	**Y**	**Y**	**Y**	**Y**	**Y**	**Y**
**3. Was the participation rate of eligible persons at least 50%?**	**Y**	**Y**	**NR**	**NR**	**NR**	**NR**	**NR**	**NR**	**NR**	**NR**
**4. Were all subjects selected or recruited from the same or similar populations?**	**Y**	**Y**	**Y**	**NR**	**Y**	**Y**	**Y**	**N**	**N**	**Y**
**5. Was a sample size justification, power description, or variance and effect estimates provided?**	**N**	**N**	**N**	**N**	**N**	**N**	**N**	**N**	**N**	**N**
**6. Were exposure(s) of interest measured prior to the outcome(s) being measured?**	**Y**	**Y**	**Y**	**Y**	**N**	**N**	**N**	**Y**	**Y**	**N**
**7. Was the timeframe sufficient to reasonably expect an association between exposure and outcome, if it existed?**	**NR**	**NR**	**NR**	**NR**	**N**	**N**	**N**	**N**	**N**	**N**
**8. For exposures that can vary in amount or level, did the study examine different levels of the exposure as related to the outcome?**	**N**	**N**	**Y**	**Y**	**N**	**N**	**N**	**NR**	**Y**	**N**
**9. Were exposure measures clearly defined, valid, reliable, and implemented consistently across all participants?**	**Y**	**N**	**N**	**Y**	**N**	**N**	**N**	**NR**	**N**	**N**
**10. Was the exposure(s) assessed more than once over time?**	**N**	**N**	**N**	**N**	**N**	**N**	**N**	**N**	**N**	**N**
**11. Were outcome measures clearly defined, valid, reliable, and implemented consistently across all participants?**	**Y**	**Y**	**N**	**Y**	**N**	**NR**	**Y**	**N**	**N**	**Y**
**12.Were outcome assessors blinded to the exposure status of participants?**	**N**	**N**	**N**	**N**	**N**	**N**	**N**	**N**	**N**	**N**
**13. Was loss to follow-up after baseline 20% or less?**	**NA**	**NA**	**NA**	**NA**	**NA**	**NA**	**NA**	**NA**	**NA**	**NA**
**14. Were key potential confounding variables measured and adjusted statistically for their impact?**	**Y**	**Y**	**N**	**N**	**N**	**N**	**N**	**Y**	**N**	**N**
**Quality rating [Good (G); Fair (F); Poor (P)]**	**G**	**G**	**F**	**G**	**P**	**P**	**P**	**P**	**P**	**P**

### Synthesis of results

#### Meta-analysis of the prevalence of youth mental health stigma

**Fig. 2:**
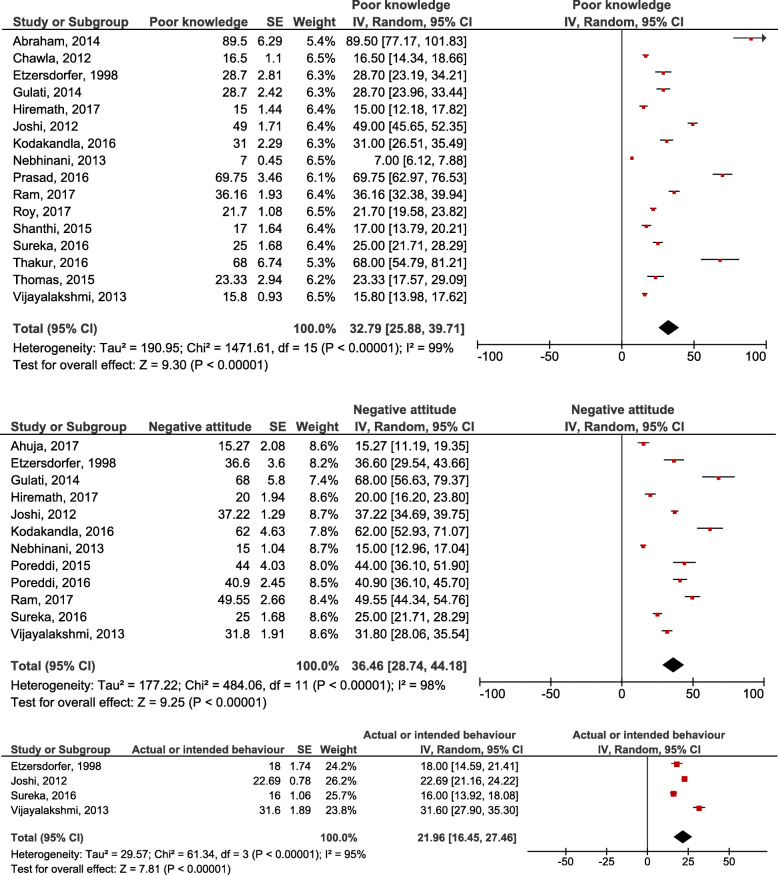
Pooled outcomes of poor knowledge, negative attitude and discriminatory intended behaviour

Percentage outcomes related to knowledge, attitude and actual/intended behaviour were pooled, as the studies were all among youth and reported similar study designs. Approximately 33% of youth participants in 16 pooled studies had poor knowledge (95% CI 25.88–39.71; *p* < 0.001), 36% in 12 pooled studies had negative attitudes (95% CI 28.74–44.18; *p* < 0.001) and 22% had stigmatising, actual or intended behaviours in four studies (95% CI 16.45–27.46; *p* < 0.001). However, this meta-analysis showed a high degree of heterogeneity, as the I^2^ value ranges from 95 to 99% (Fig. 2).

#### Gaps in conceptualising mental illness

In order of frequency and importance across 21 included studies, Table 4 presents a summary of gaps corresponding to each stigma component: knowledge, attitude and behaviour.

**Table 4:** Characterizing mental-health-related public stigma: common conceptual gaps and perceptions among Indian youth

**Component of stigma**	**Themes and sub-themes**	**Frequency of the theme in study results** (≤2 = Small (S), 3–5 = Medium, > 6 = High)	**Number of studies reporting on the theme in results**	**Included in the discussion**
**Knowledge**	***Symptoms/ expected behaviour of a person with a mental illness***	H	8 [34, 42, 44, 45, 52, 54, 60, 62]	2 [45, 52]
	Low I.Q.	M		
	Difficult to identify	M		
	Likely to harm others/ violent	M		
	Unpredictable behaviour	S		
	Withdrawn/ passive	S		
	Unable to manage emotions	S		
	Life satisfaction	S		
	Speak differently	S		
	Differences in sleeping, eating and memory	S		
	Multiple personalities associated with depression	S		
	***Treatment and recovery***	H	7 [34, 36, 37, 44, 45, 54, 57]	4 [34, 36, 44, 57]
	Unlikely to be cured (recurrent, lifelong)	M		
	Marriage as a social intervention	M		
	Treatment by a spiritual healer	S		
	Heavy/ multiple medication/ hospitalisation needed	S		
	***Causes of mental illness***	H	3 [34, 44, 57]	5 [34, 42, 44, 57, 62]
	Genetic/ hereditary/ birth defect	M		
	Evil spirits and bad deeds	M		
	Brain damage	S		
	Stress	S		
	Social environment	S		
	Infectious transmission	S		
	Physical/ sexual abuse	S		
	Non-vegetarian diet	S		
	***Alcohol and its effects***	M	4 [40, 49, 55, 56]	3 [40, 49, 55]
	Associated with temporary harm	M		
	Locations to access	S		
	Prevalence of youth substance use	S		
	Treatment/ cessation services	S		
	Associated with suicide	S		
	***Can happen to anyone***	S	2 [54, 60]	–
	Mental illness can happen to anyone	S		
	Suicide happens to few people	S		
	Suicide occurs more among women	S		
**Attitude**	***Cannot shoulder responsibility or take life decisions***	H	6 [34, 45, 50, 52, 57, 60]	7 [34, 42, 45, 50, 52, 57, 60]
	Should not marry or should be married			
	Unlikely to be good parents	S		
	Unable/ incapable of having a job	S		
	Cannot take decisions in their own treatment	S		
	Voting	S		
	Poor interpersonal or social skills	–		
	***Dangerous***	M	4 [44, 45, 52, 60]	4 [41, 42, 45, 52]
	Likely to be violent	M		
	Likely to commit crimes (need punishment to prevent future attacks)	S		
	Intolerant of suicidal ideation	S		
	***Avoid people with mental illness***	M	3 [42, 44, 60]	1 [42]
	Desired physical separation (should be treated in different hospitals from people with physical illness, kept locked, in special schools)	M		
	***Negative emotions***	H	8 [34, 41, 42, 44, 45, 48, 57, 60, 61]	5 [34, 41, 42, 45, 52]
	Shame and blame (cowardly, inferior, lacking will power, should not	H		
	disclose illness, deliberately acting so)			
	Fear	S		
	Pity/ sadness	S		
	Low status of psychiatry/ psychiatrists	S		
	Attention-seeking	S		
	***Substance use***	M	3 [46, 49, 55]	2 [49, 55]
	People who use are ‘bad’	S		
	Improves sexual activity	S		
	Proximity to users increases risk of substance use	S		
	Alcohol as a status symbol/ celebratory product	–		
	Use is common in social scenarios	–		
**Behaviour**	***Treatment***	M	2 [44, 57]	2 [44, 57]
	Not taking a person with an epileptic seizure to the hospital, throwing	S		
	water on them or making them smell a shoe			
	Treatment should be separate from physical problems or confinement	S		
	***Personal interactions***	S	2 [34, 60]	2 [34, 42]
	Not maintaining friendships	S		
	Laughing at persons with mental illness	S		
	***Help-seeking for self***	S	1 [41]	2 [52, 60]
	Commit suicide if diagnosed with a mental illness	S		
	Not disclose own mental illness	S		

### Knowledge

A significant majority of participants in some studies believed that people with a mental disorder can never recover [36, 37, 45]. One study suggests that in the Indian context, social distance was determined to a greater degree by lack of knowledge about recovery rather than perceived unpredictability or dangerousness [36]. Unsurprisingly, youth perceived that a battery of allopathic, ayurvedic and homeopathic treatment was required to treat mental illness, [57] or that control over symptoms was possible only with prescription drugs [34] or hospitalisation [54]. Youth in other included studies believed that mental illness was principally due to genetic or supernatural causes, [34, 44, 57] or believed in myths that mental illness is infectious or due to a non-vegetarian diet [44]. Only one study from the capital city, Delhi found that environmental factors such as stress, biological factors and physical and sexual abuse, were perceived causes of mental illness among youth [34]. As a result, it is plausible that believing that factors outside of one’s individual and social control are responsible for mental health problems may be linked to beliefs that interventions to alleviate such problems are also beyond one’s control. Further, although youth believed that it was easy to recognize people with mental illness when compared to people who suffer from other physical illnesses, [42, 60] they were not able to correctly identify symptoms of mental health problems in any studies (including linking alcohol with only temporarily harmful effects) [49, 54–56].

### Attitude

Negative perceptions that people living with mental illness are unable to control the problem, and are likely to be dangerous, violent, criminal or unpredictable was held by more than 70% of youth in four studies reporting these outcomes [44, 45, 52, 60]. Beliefs that people with mental illness are cowards, [41] lack willpower, [49] are difficult to like [64] and are to blame for their problems [36, 37] were found in several studies. Other studies found that people with mental illness were assumed to be less intelligent than others, [44, 60] or be prone to changing their mind quickly [54]. In a study, suicide was perceived as a cowardly act by 70% of youth, 29% said it was impulsive and 36% said it was deliberate [41]. Talking about suicide was perceived to increase the risk of suicide [54] and since youth believed that people who are serious about suicide do not talk about it, [48] youth would likely find it difficult to communicate about such problems and brush aside disclosure of suicidal intent. In contrast, 89% of the students felt pity for an epileptic patient in one study [44]. A study showed that attitudes were most positive towards people with intellectual disability, and less favourable to people with acute mental illness, and least of all towards people who were associated with substance misuse [46]. Where substance use was involved, people with mental illness were labelled ‘bad’ and were expected to overcome their problem through will power [49].

The responsibility of work and social roles was deemed too difficult for people with mental illness by 41%, [52] 71% [50] and 63% [45] of youth in three studies. A study suggested that youth believed that people with mental illness could only be given work with minor responsibilities [45]. However, youth were divided between whether people with mental illness should get married and have children, as a form of treatment of their illness. In another study nursing students felt that mental illness was a strong ground for divorce compared to business management students, although business management students held significantly more stigmatising views than nursing students on whether people with mental illness should have children and hold a job [60]. Overall, studies show that youth were unaccepting of the autonomy and independence of theose suffering from mental illness and did not consider them capable of managing their personal and professional life.

### Intended behaviour

Social distance [36] and stigmatisation [51] were likely behaviours of Indian youth towards people with mental health problems. Most youth in studies preferred to exclude people with mental health problems from treatment-related decision-making [50] and education [44]. In one study, between 25 and 40% of health professionals in-training believed that people with mental health problems need to be separated from others with physical illnesses for treatment [42]. Youth in a study would prefer to lock up or punish people with mental illness, out of fear of being attacked [42]. A third of business management students were significantly more likely to move out of a neighbourhood if a mental health facility was set up compared to nursing students [60].

About 48.5% of students in a study would not take a person suffering from a seizure to the hospital [44]. Unusual and shame-inducing practices, such as making the person smell a shoe or an onion, were associated with likely behaviours of youth towards a person going through an epileptic attack [44, 57]. Youth in two studies preferred not to disclose mental illness, [41, 60] with nearly 20% of youth in one study reporting that they would likely commit suicide if they developed a mental disorder [41]. However, in one study youth believed that feeding and keeping people with mental illness comfortable, equivalent to ‘throwing money at the problem,’ was not enough [42].

### Recommendations to reduce youth mental health stigma in India

#### Content and terminology

Most studies identify the need for interventions to sensitize students about potential causes, treatment effectiveness and duration, and abilities of people living with mental illness [33, 34, 44, 46, 50, 55, 57, 60, 62]. A study suggested that using lay language and commonplace perspectives on mental health and community-based interventions may aid in reaching more youth [62]. Moreover, the use of bio-medical explanations and terms was found to intensify discriminatory attitudes [62, 65]. Studies by Bell emphasize that the authors made assumptions that a common understanding of schizophrenia and severe depression exists, whereas participants may have understood survey instruments differently [37]. Finally, a study also hypothesized that emphasizing mental ‘fitness’ or wellbeing as a goal of mental health promotion may be more appealing and acceptable to youth [62].

#### Integrating with educational curriculum

Information campaigns targeting youth and the general public are emphasised as a key step towards reducing mental health stigma [44, 45, 49]. Public health awareness programs that use a broad, behaviour-focused approach were recommended to improve suicide and depression literacy [54]. As many studies in this review evaluate the level of stigma among health professionals in-training, enhancing educational curriculum, professional ethics and code of conduct, awareness camps and clinical training for improved treatment and care practise are advocated [38, 42, 45, 52, 57, 61]. Some strategies to inculcate positive attitudes among medical and nursing students include: short educational interventions, [51] participation by consumers, [66] and use of role play and entertainment-education techniques [43]. In educational settings, recommended initiatives to reduce stigma include: continuous and repetitive educational efforts in partnership with parents and teachers, [44] reaching students who are not necessarily in direct contact with mental illness, [60] and lectures, media and wider campaigns about treatment of substance abuse [49]. Further, youth volunteering in activities or programs related to mental health may help them to build skills in mastering their environment [62].

## Discussion

The most notable gap related to knowledge of mental health problems among young people in India was that all such problems were considered to be acute, severe or serious and therefore, people with such problems are perceived as dangerous or unable to manage their daily life or function as per societal roles and norms. Although US and Latin American youth perceive people with mental health problems as more dangerous if they associated these problems with genetic causes or biological reasons, [16, 23] Indian youth lack knowledge about causes and largely associated such problems with functional impediments and believed that limited/no treatment exists for such problems. Next, young people in included studies were both unable to identify common symptoms or use a common term or psychiatric label to describe symptoms. Consequently, young people in India may not consider themselves vulnerable to acute problems or recognise every day mental health problems when they experience them. Similar to a cross-sectional survey of public stigma among 15–60 year old Indians, [67] this review found that neither symptoms nor psychiatric labels nor mental illness (broadly) are widely recognized or understood. Since different expressions and thresholds for accepting symptoms of mental health problems may lead to such problems often going unnoticed [10, 68] and since psychiatric labels may potentially induce prejudice (e.g. ‘depressed’ was self-rated as derogatory), [69] there is a need for culture-specific explanatory models of mental illness or use of culturally-appropriate vignettes instead of focusing on psychiatric labels to aid young people in recognizing mental health problems from an early age, in both stigma-assessment research questionnaires [70] and anti-stigma communication strategies [7, 71, 72].

Recommendations to reduce stigma by studies in this review include implementation of de-stigmatization and information-sharing interventions to build awareness and sensitize youth about mental health problems. Unlike, high income countries with national mental health education and promotion campaigns, such as the Time to Change campaign, [73] and Headspace, [74] India has no such country-wide mental health awareness campaign. Recent anti-stigma programmes involve university students in peer-led educational components as in the Active Minds and University Bring Change to Mind programmes [75, 76]. A community-based anti-stigma campaign in India improved attitudes and intended behaviour towards people with mental health problems; however, it lacked a control group and targeted people above 18 years of age [77]. In the future, such interventions may be adapted to appeal to young people to address their age-appropriate needs and communication issues. Thus, future anti-stigma interventions should integrate with the education system, use interactive/ visual media and focus on mental health problems broadly, by defining and explaining symptoms through relevant vignettes or stories, rather than using psychiatric labels for specific disorders or illness.

This review shows that public stigma among youth in India has similar characteristics to public stigma in other cultures. Studies in this review show that Indian youth expressed fear, shame, sadness, pity or sympathy, similar to global attitudinal responses of ‘stigmatisers.’ [78] As in studies from Lebanon, [79] Singapore [13] and China, [80] evil spirits and God’s punishment were important determinants of public stigma in India relative to environmental factors. Similar to adolescents in Greece, [81] Indian youth believed that mental health problems were easily identifiable and that people with such problems appeared markedly different. We also find that considerable youth believe in both traditional/faith healers and psychiatry as part of India’s pluralistic medical system [82, 83]. Additionally, our findings resonate with other studies that marriage and child-bearing are important life events, which represent social worth in Indian and Asian culture, [84–86] unlike in Western countries [7], but Indian cultures likely differ from the West in that autonomy, decision-making and capability of young people with mental health problems are overruled by adults. Further, culture may alter how participants perceive mental health problems, for example, alcohol consumption may not be perceived as harmful because of traditionally acceptable use of some addictive substances (e.g. betel nut) in India.

Potential factors that likely exacerbate stigma in India are that people in Asian cultures accept and observe status inequality more readily, [87] and youth seek to satisfy adults and echo the views of their families; a collectivist identity, where people fear what that others know about their problems [88] and gender inequality, since global studies report higher social distance among females than males [89–91]. Thus, despite the belief that Indian culture has protective, cohesive family environments which has the potential to readily accept those suffering from with mental health problems, [68] mental-health-related stigma persists. A study of 11 countries comparing how stigma operates in the East and West found that ‘deep cultural concerns about how being diagnosed with a mental illness would impact family members,’ social and economic status,’ fear of disclosure and moral attributions affected stigmatizing attitudes in Eastern countries [92]. Such findings may also apply to youth in India and other low- and middle-income countries where there is a lack of understanding about stress and mental health issues, which then interact with other issues such as coping with poverty, in addition to strong cultural beliefs.

### Strengths and limitations

This is the first systematic review to collate findings from mental -health -related stigma studies focused on youth in India. There are no other country-specific, youth-focused systematic reviews and meta-analyses on public stigma. The approach of assessing the magnitude of stigma and method developed are also unique to this review. This review outlines the evidence for an age-appropriate educational response to reducing public stigma in India, in three key ways: (i) quantifying the problem and the rationale for change; (ii) identifying and characterizing common gaps in knowledge, attitude and behaviour that require counter-messages; and (iii) synthesizing strategies to reduce public stigma. By applying the method used in this review, future studies may compare characteristics of youth stigmatization of people with mental health problems across countries and cultures. We believe that lectures, talks and discussions suggested by studies in this review may work for health professionals in-training, who develop stigma in a unique way, [93] however, alternative approaches will be required to engage students pursuing other disciplines who lack exposure to information about mental health problems and could have perhaps not previously encountered a person with a mental health problem. Such approaches must focus overtly on challenging stereotypes, by including more visual-based interaction and relatable language.

Although results of the meta-analysis present a worst-case scenario, selecting negative responses only, it highlights the magnitude of mental -health -related stigma and the need for intervention among youth in India. Potential reasons for high heterogeneity among pooled studies include varying definitions or terms, a range of assessment measures to gauge stigma and use of non-standard data collection procedures. Given the limited number of studies providing adequate information on stigma, it was not feasible to assess whether stigma associated with particular disorders/conditions was similar to that of stigma associated with mental illness more generally or other disorders. As more than half of the included studies had a fair risk of bias and pooled data showed high heterogeneity, the review findings are unlikely to be valid among youth in other settings in India. Further, a lack of studies among school-going adolescents skew our results towards college youth and particularly, health professionals in-training. The quality of stigma-related studies may be improved in future cross-sectional studies through randomised sampling and sample size estimation, use of validated instruments and improved reporting. Due to lack of age-segregated data in community-based knowledge, attitude, behaviour–assessment studies, a comparison between the level of public stigma between Indian youth and adults was not feasible. As studies in this review were skewed by geography and population groups, it was not feasible to identify specific youth groups or regions which could be targeted to reduce public stigma. In addition, since most studies used survey instruments designed for adults, marriage and child-bearing find greater mention than education, employment, friendships or other youth-relevant milestones. Finally, one article was unavailable for inclusion in this systematic review.

To update this review with the most recent studies, we conducted the search strategy in PubMed and CINAHL+ (for the period September 2018–2020). Since 2018, we found six additional studies (including two that were previously unavailable), all of which support findings presented in this review. A quantitative study using a new scale found 18–24 year-old Indians’ attitudes to suicide as negative, and that they felt suicide could not be prevented and that there were no risk signs [94]. Two other studies found poor levels of knowledge, with one study showing that 53.7% of students had poor knowledge regarding preventive measures of suicidal behaviour [95] and another showing that 43% of school students had inadequate knowledge of substance use [96]. Another study found that medical interns agreed that ‘patients like this (with psychiatric illnesses) irritate me’ or treating them was a ‘waste of money.’ [97] A qualitative study echoed our findings that mental health and mental illness were unclear concepts and were associated with acute problems, such as ‘brain deficiency or dysfunction and abnormal behaviour.’ [98] Another qualitative study found that college students believed that using substances helped to relieve depression, enhance health and lose weight and that using in small quantities did not cause harm [99]. The study suggests that future interventions should be non-judgemental, student-friendly, relatable and ‘specific to the youth’s life circumstances and needs.’

Notably, updating the systematic review also highlighted several studies that contribute to the social context of mental-health-related stigma. A qualitative study of community stakeholder perspectives (not including youth) described that schools are hesitant to acknowledge the extent of mental health problems and students fear being labelled, thereby creating an environment of hiding mental health problems [100]. The study also highlighted the need for school and college counsellors and mental health training for teachers. Our review includes studies on nomophobia, an emerging mental health issue, which is echoed by contextual studies that finding increasing rates of substance use and technology addiction among youth due to urbanization in India [101]. Other studies focused on measuring prevalence, progression perceived harms of various disorders and conditions, including depression, anxiety and stress, [102, 103] alcohol use, [104, 105] body image disorders [106, 107] and aggression, bullying and violence, [108] and correlates such as parental pressure to perform academically, [109, 110], relationships, negative peer pressure, school environment and gender roles [110]. [111].

## Conclusions

India is home to a third of the world’s youth. Mental health problems are likely to adversely impact the productivity and capabilities of India’s youth. Among youth included in this review, one-third had poor knowledge and negative attitudes, and one-fifth intended to or had actually discriminated against a person with mental illness. Although most of these studies were among college students, they were predominantly focused on health professionals in-training. A majority of youth potentially recognized mental health problems only if they were acute. Select aspects of traditional Indian culture, such as importance of marriage, are likely responsible for specific manifestations of stigma. Educational interventions to reduce stigma associated with mental health may improve help-seeking behaviours by avoiding the use of psychiatric labels that are not commonly understood, instead focus on symptomatic vignettes that may explicitly discuss a range of mental health problems with varying severity. Intervention content that directly and interactively discusses youth mental-health-stigma-related responses and age-appropriate social roles, rather than focusing on future roles such as marriage, may help to achieve timely detection of mental health disorders among youth.

## Supplementary Information

**Additional file 1.**

## Data Availability

The data supporting the conclusions of this article are included within the article tables and figures.

## Abbreviations

PRISMA: Preferred reporting items for systematic reviews and meta-analyses
SE: Standard error
CI: Confidence interval
ESRC: Economic and social research council
K: Knowledge
A: Attitude
B: Behaviour
